# Supplementary material for: Na+/H+ Exchangers Involve in Regulating the pH-Sensitive Ion Channels in Mouse Sperm
Source: Int J Mol Sci. 2021 Feb 5;22(4):1612. doi: 10.3390/ijms22041612 (PMC7914462; doi:10.3390/ijms22041612)
Supplement: Supplementary file 1 [file ijms-22-01612-s001.zip › Supplementary file.docx]

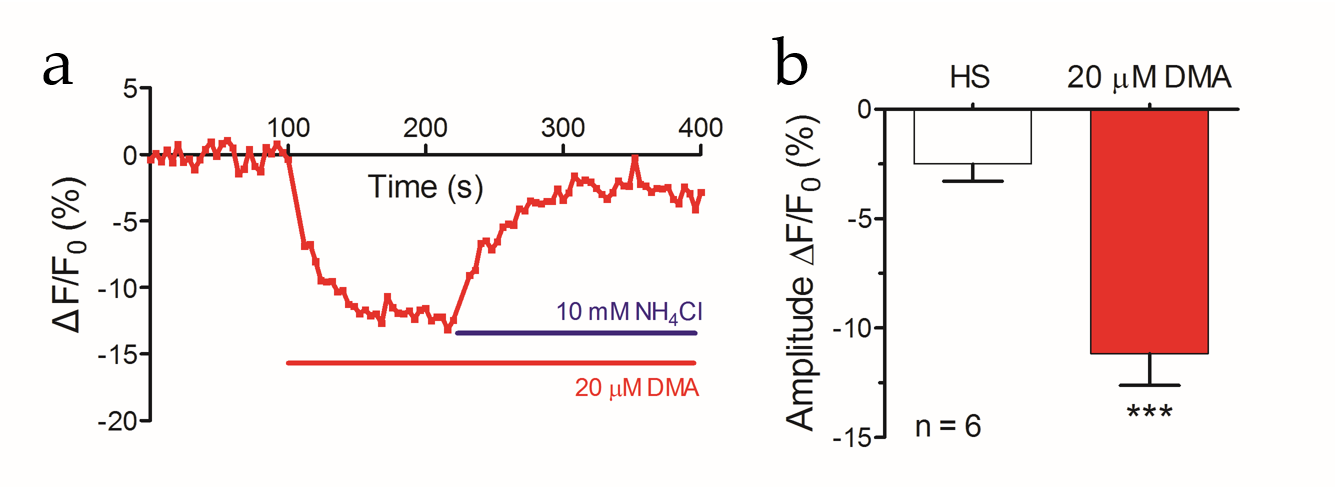


Supplementary Fig. 1 pH_i_ in mouse sperm was reduced by DMA application. (**a**) The fluorescence time-course trace representing the changes in pH_i_. NH_4_Cl-evoked pH_i_ changes served as a positive control. Bottom, DMA (20 μM) acidified the cytoplasmic pH_i_ of sperm. △F/F_0_ (%) indicates the percent change in fluorescence (△F) with respect to the mean basal fluorescence (F_0_). (**b**) Mean amplitudes in the absence and presence of 20 μM DMA. Data are expressed as mean ± SEM. n = 6 (***P < 0.001, paired *t*-test).


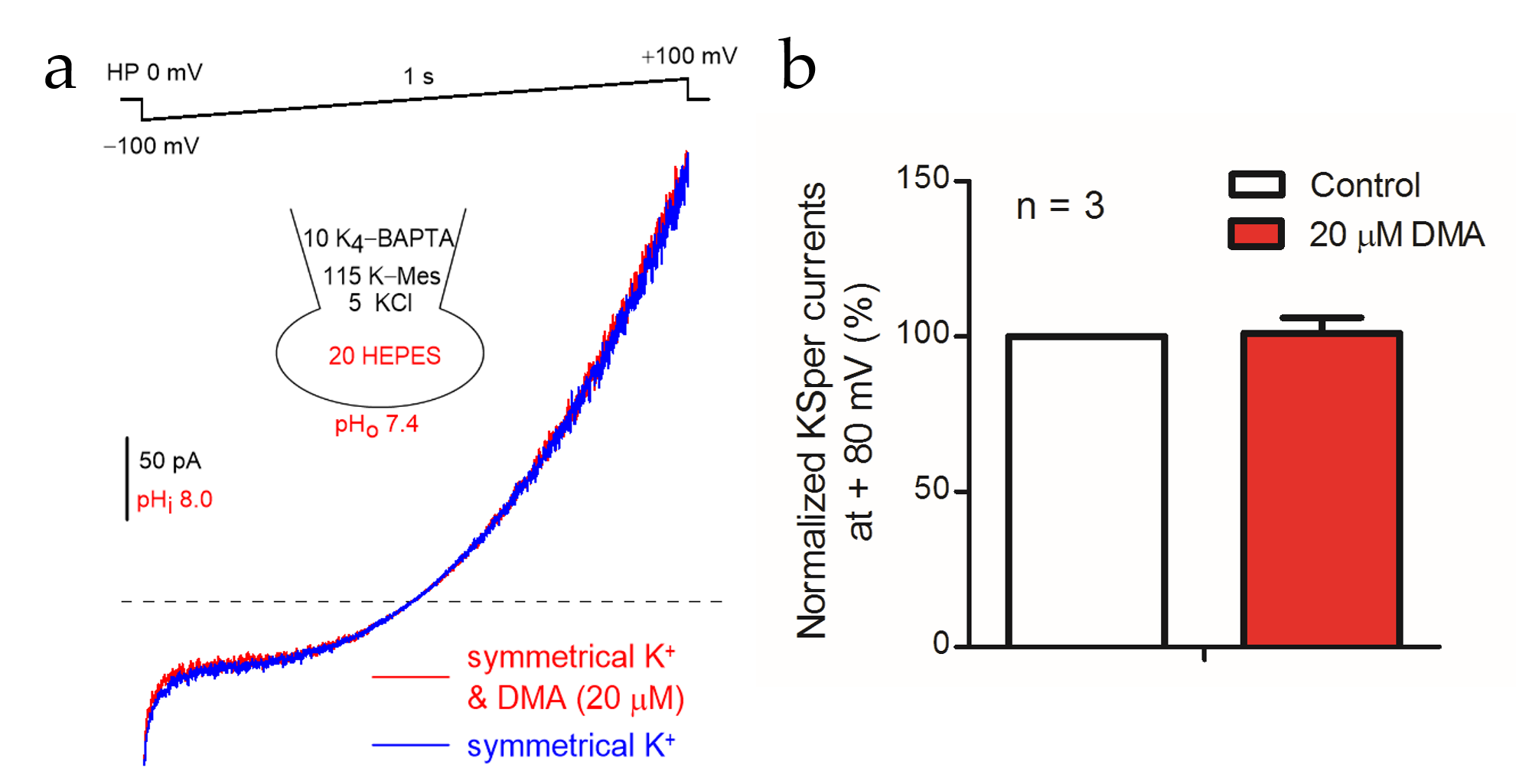


Supplementary Fig. 2 KSper channel was not affected by DMA. (**a**) Representative patch-clamp recordings of KSper current recorded in the symmetrical K^+^ solution with the pipette solution containing pH buffer at pH_i_ 8.0. (**b**) Mean amplitudes of normalized KSper current at + 80 mV before and after the perfusion of 20 μM DMA. Data are expressed as mean ± SEM. n = 3.


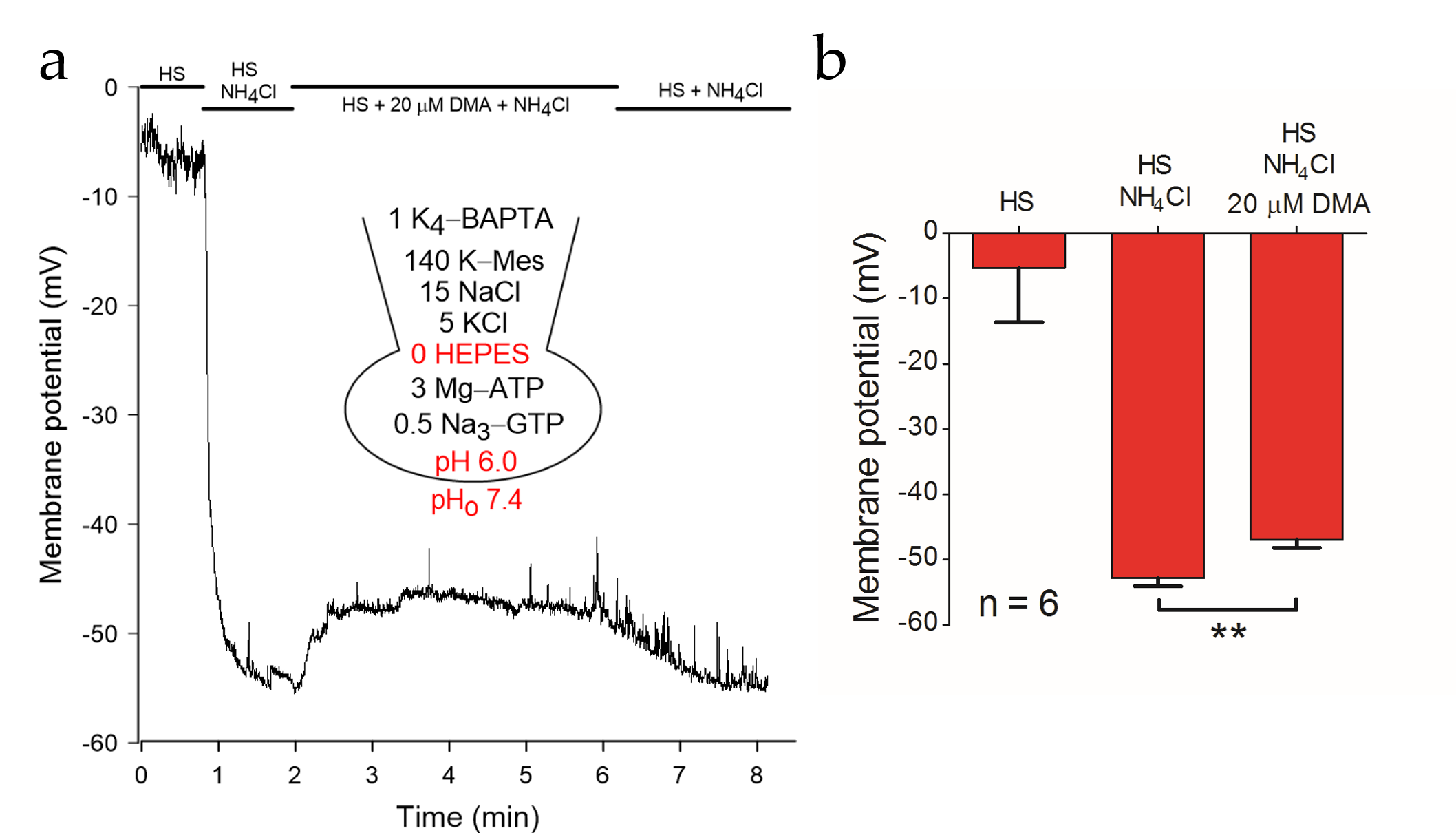


Supplementary Fig 3. The alkalization-induced hyperpolarization was reversibly weakened by DMA application in a pH 6.0 pipette solution without pH buffer. (**a**) The effect of DMA on the NH_4_Cl-induced hyperpolarization at pH_i_ 6.0 was shown. (**b**) Mean amplitudes of membrane potential at pH_i_ 6.0 in the presence of 10 mM NH_4_Cl before and after the perfusion of DMA. Data are expressed as mean ± SEM. n = 6 (**P < 0.01, One-way ANOVA).


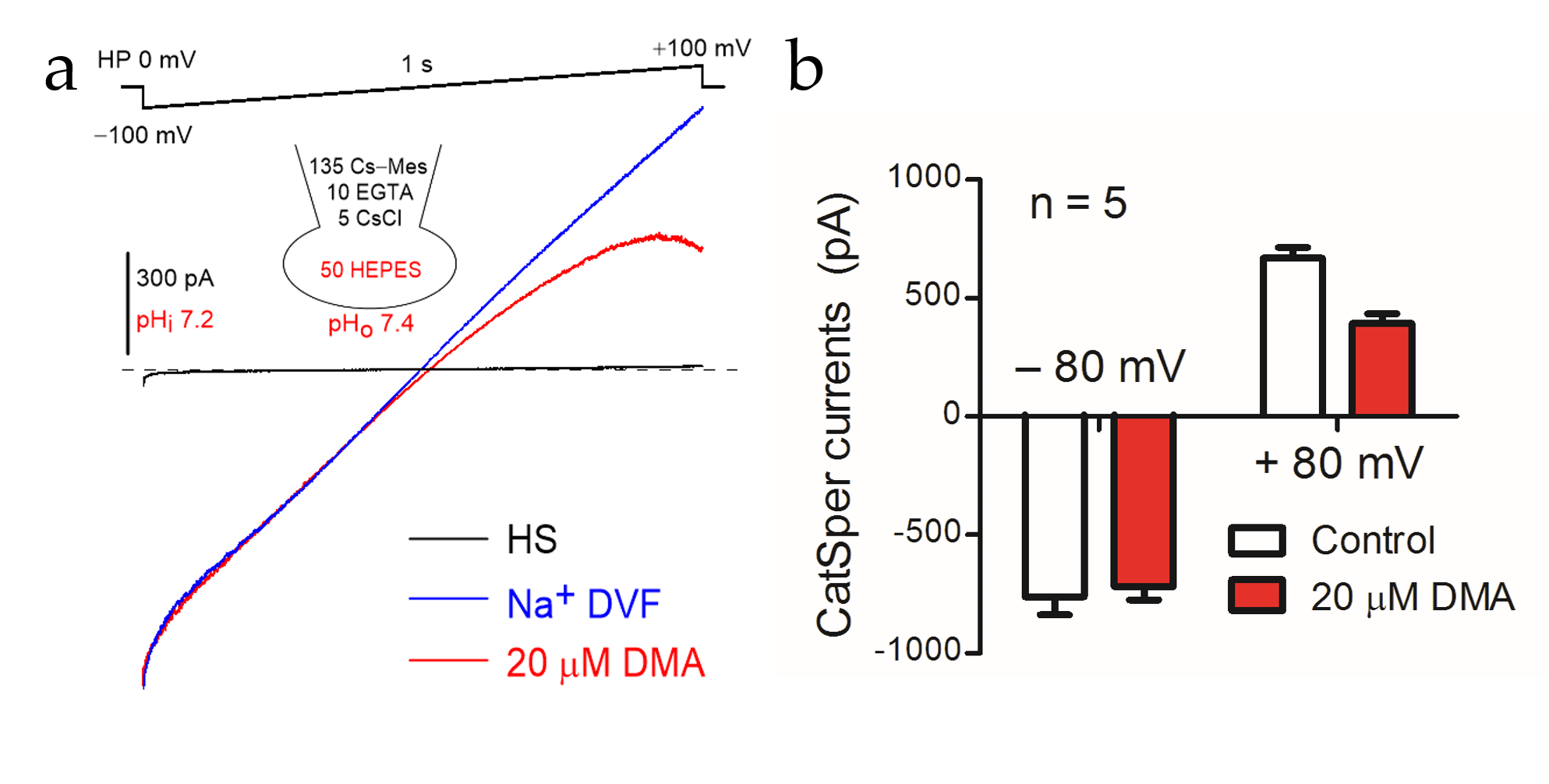


Supplementary Fig 4. DMA inhibited the outward current of CatSper with a strong pH buffering in the pipette solution. (**a**) Representative patch-clamp recordings of CatSper current in mouse sperm in the absence or presence of DMA elicited by 1s voltage ramp from – 100 mV to + 100 mV with a pipette solution containing 50 mM HEPES at pH_i_ 7.2. (**b**) Statistical analysis of the mean CatSper currents in the absence or presence of DMA at – 80 mV and + 80 mV. Data are expressed as mean ± SEM. n = 5.


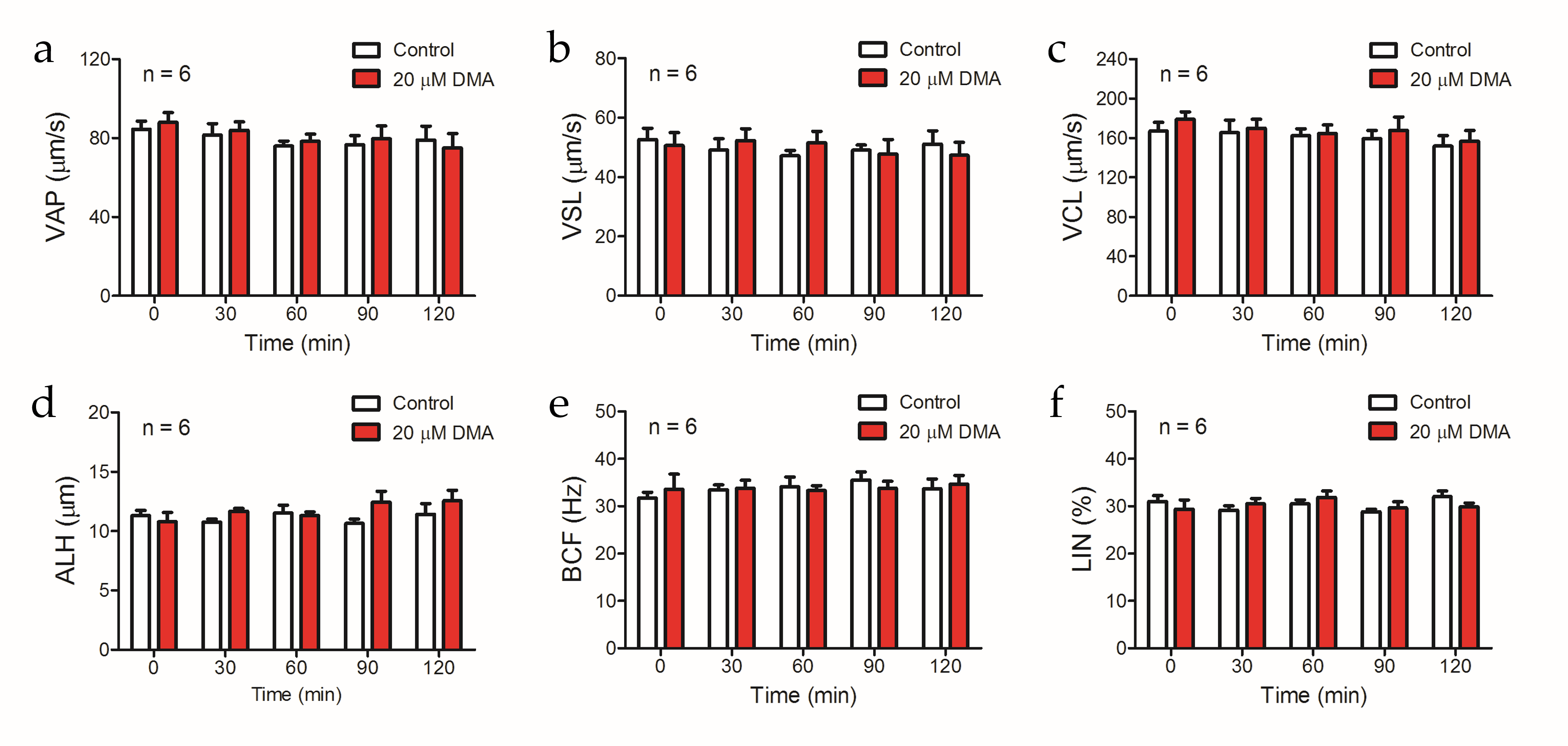


Supplumentary Fig 5. Sperm motility parameters were not affected by DMA. The change of VAP (**a**), VSL (**b**), VCL (**c**), ALH (**d**), BCF (**e**) and LIN (**f**) after the incubation of DMA at 0, 30, 60, 90 and 120 min was shown. Data are expressed as mean ± SEM. n = 6.


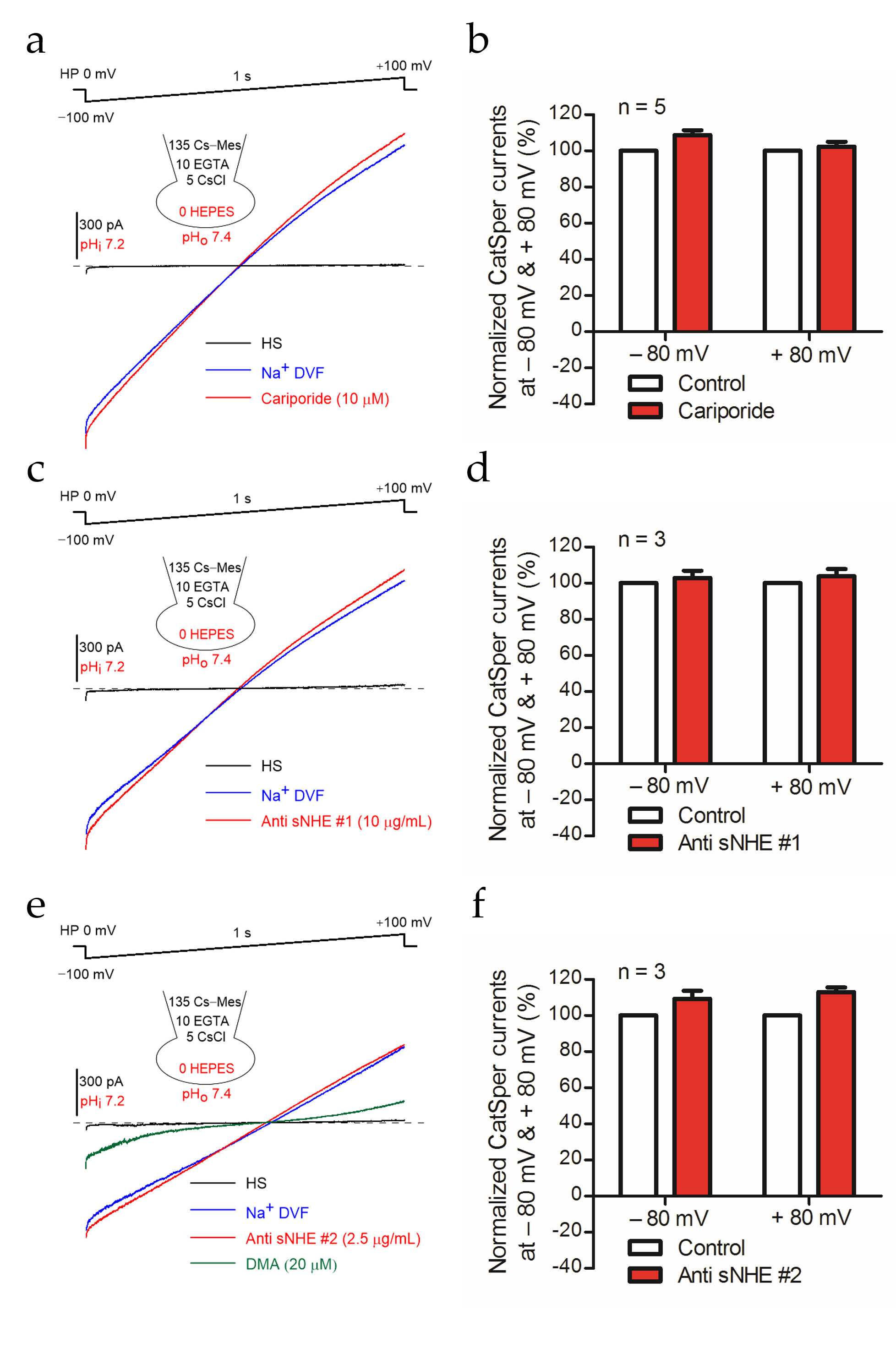


Supplementary Fig 6. (**a**) The effect of cariporide on the CatSper current in the pipette solution without pH buffer (0 mM HEPES). (**b**) The statistical results of normalized CatSper current in the presence or absence of cariporide related to (**a**) was shown. n = 5. (**c**) The effect of Anti-sNHE #1 on the CatSper current in the pipette solution without pH buffer (0 mM HEPES). (**d**) The statistical results of normalized CatSper current in the presence or absence of Anti-sNHE #1 related to (**c**) was shown. n = 3. (**e**) The effect of Anti-sNHE #2 on the CatSper current in the pipette solution without pH buffer (0 mM HEPES). DMA was applied as the positive control. (**f**) The statistical results of normalized CatSper current in the presence or absence of related to (**e**) was shown. n = 3. Data are expressed as mean ± SEM.
